# Genotypic variation in agronomic and physiological responses of potato cultivars to water stress under greenhouse conditions

**DOI:** 10.3389/fpls.2025.1692962

**Published:** 2025-10-15

**Authors:** Fadia Chairi, Marc Lateur, Yordan MuhovskI

**Affiliations:** ^1^ Department of Life Sciences, Biodiversity and plant and forest improvement unit, Walloon Agricultural Research Center, Gembloux, Belgium; ^2^ Department of Life Sciences, Biological engineering unit, Walloon Agricultural Research Center, Gembloux, Belgium

**Keywords:** potatoes, drought stress, physiological traits, stomatal conductance, genotypic variation

## Abstract

Due to the effects of climate change, conditions tend to be increasingly extreme, with water availability being one of the main limiting factors in potato production. The objective of this study was to analyze the differential response of physiological and yield components in eight potato varieties under water deficit conditions. For this purpose, a greenhouse trial was carried out with the varieties Bintje, Montis, Sevilla, Lady Jane, Louisa, Dior, Acoustic and Tentation. Varieties were submitted to a moderate water stress (MS) and severe water stress (SS) from 23 days after planting (DAP) until 55 DAP and compared to well-watered (WW) condition. Data were collected on morphological traits such as plant height and root length and weight. As well as the physiological traits such as stomatal conductance (gs), chlorophyll and Flavonoid content, relative water content (RWC), water use efficiency (WUE) and carbon isotope composition. At 55 DAP yield components were determined. Variation of all parameters (Δ) in comparison with control were calculated and contrasted with a drought tolerant index (TDI). Drought stress significantly reduced biomass, tuber number, and plant height, but genotypes responded differently, reflecting notable genetic diversity in tolerance mechanisms.

## 1 Introduction

Potato (*Solanum tuberosum L.)* is the most important non-grain crop in the world and the third most important food crop, regularly consumed by billions of people producing more than 376 million tons of tubers and yielding 20.7 t/ha in 2021 . However, its productivity is limited by degradation of soil root zone and the consequences of climate change such as drought and heat (IPCC 2021). According to Anithakumari et al. (2012) and Haverkort and Verhagen (2008), potato plants are drought-sensitive due to their short and shallow root system, which can result in considerable losses in production and tuber quality.

Globally, the frequency and severity of drought have increased due to the varied precipitation and rising temperature. This trend poses a significant challenge to potato production. Drought, characterized by prolonged periods of insufficient rainfall, induces severe water stress in plants, which can affect their key physiological processes and overall growth (Monneveux et al., 2014). Indeed, they affect not only yield, number, and size of tubers (Haverkort and Struik, 2015) but also the quality of the tubers produced by increasing defects such as cracking and malformation and decreasing dry matter content (Aliche et al., 2018; Alvarez-Morezuelas et al., 2022).

Moreover, drought stress leads to physiological changes, including alterations in stomatal conductance, leaf water content, and photosynthetic efficiency (Farooq et al., 2009). Understanding these physiological responses has become a key issue for their potential application in breeding programs aimed at enhancing climate resilience in potatoes.

Among these parameters, stomatal conductance is an essential process for photosynthesis and transpiration, as it regulates gas exchange and water loss (LE et al., 2011). During drought, plants often exhibit reduced stomatal conductance, a water conservation mechanism, which can lead to decreased photosynthetic rates and, consequently, reduced growth and yield (Saravia et al., 2016; Shoukhat and Tutail, 2025). The relationship between stomatal conductance and drought stress is complex, as it involves trade-offs between water conservation and the plant’s ability to assimilate carbon dioxide for photosynthesis. Besides stomatal conductance, other physiological parameters, such as relative water content (RWC) and chlorophyll fluorescence, are essential for assessing the impact of drought on potato plants. RWC measures the water status of plant tissues and serves as an indicator of plant hydration and stress. A decrease in RWC is often correlated with an increase in water stress, leading to impaired physiological functions (Alvarez-Morezuelas et al., 2022). Chlorophyll fluorescence, on the other hand, provides information on the efficiency of photosystem II, allowing researchers to assess the photosynthetic performance of plants under varying environmental conditions. Changes in chlorophyll fluorescence parameters can indicate the extent of photoinhibition and stress experienced by plants during drought (Farooq et al., 2009).

Research has shown that some potato varieties exhibit specific responses to water deficit through changes in RWC, leaf water potential, stomatal resistance, transpiration rate, and temperature at the leaf and canopy levels (Ahmadi et al., 2010). In potato, water stress results in a reduction in stomatal conductance when leaf water potential falls below −0.6 MPa and is often accompanied by a decrease in photosynthetic rate, typically between 1 and 3.6 μmol CO_2_ m^-^² s^-^¹ under controlled greenhouse conditions (Vasquez-Robinet et al., 2008). In addition to changes in water-related parameters, water deficit can also affect leaf chlorophyll content. Under mild to moderate water restriction, chlorophyll concentration per unit leaf area often increases due to reduced leaf expansion, which concentrates pigments in a smaller tissue volume (Rolando et al., 2015).

A better understanding of the physiological responses of potato plants to drought, particularly in terms of stomatal conductance, RWC, and chlorophyll fluorescence, is crucial for developing effective strategies to enhance drought resilience. As the agricultural sector continues to grapple with the challenges posed by climate change, research focused on the intersection of drought stress and potato physiology will be vital in ensuring the sustainability of this important crop.

The identification of drought-tolerant potato genotypes could be a good strategy for mitigating the effects of climate change on the productivity of this crop. In this context, the present study aimed to investigate the differential responses of eight potato varieties by evaluating selected physiological traits and yield related parameters f under water stress conditions in the greenhouse.

## 2 Materials and methods

### 2.1 Plant material and growing conditions:

The study used 8 potato (*Solanum tuberosum L*) genotypes, all commercial cultivars (**Table 1**). They were selected for their different range of productivity and maturity.

**Table 1:** Set of eight potato cultivars tested in this study.

Cultivar	Year of release	Country	PEDIGREE	Breeder
Acoustic	2018	NL	ORCHESTRA x DOB1997-507-015	Meijer
Dior		NL	AKTIVA x LAPERLA	Den Hartigh
Montis	2020	DE	ISP 19-8–03 x ISP 14-15-03	Interseed Potatoes Gmbh
Tentation	2015	FRA	(ALTESSE x EMRAUDE) x COQUINE	Grocep (F) Van Rijn France (F)
Louisa	2017	BE	GASORE x VICTORIA	CRA-W
Bintje	1910	NL	MUNSTERSEN x JAUNE D’OR	K.L. DE VRIES
Sevilla	2018	NL	AGRIA x DOB1997-507-015	Niek Vos
Lady Jane	2020	NL	AGRIA x CMK 05-709-005	Meijer

Year of release, country of registration, pedigree and breeder are presented.

The experiment commenced on 16 May 2023 in a greenhouse at the CRA-W experimental fields in Libramont, Belgium, using a Randomized Complete Block Design (RCBD) with three replications per treatment (control well-watered, WW, moderate water stress, MS, severe water stress, SS) for each genotype. The greenhouse conditions were maintained at a temperature of 22 ± 2 °C during the day and 16 ± 2 °C at night, with a photoperiod of 16 h light/8 h dark and an average light intensity of 400 μmol m^-^² s^-^¹.

Plants were growing in plastic pots (7.5 L) filled with a commercial potting soil. Each pot contained one plant, and three pots per genotype were used for each treatment. A layer of vermiculite was placed on the soil surface to minimize evaporation.

After initial planting, plants were irrigated to maintain 100% water capacity. Starting from 7 June 2023 (23 DAP), the stress treatments were implemented by withholding irrigation until 13 June 2023 (30 DAP). At that point, the conductance capacity of the most sensitive plants reached 50 mmol H_2_O m^-^² s^-^¹, and water (400 mL for moderate water stress and 200 mL for severe water stress) was added every 2 days to mimic varying levels of drought stress. Pots were weighed before irrigation to calculate water use (evapotranspiration).

### 2.2 Eco physiological parameter

#### 2.2.1 Leaf pigments

The content of different leaf pigments per area basis was assessed at 26, 29, 31, 35 and 50 DAP using a portable leaf-clip sensor (Dualex, Force-A, Orsay France), which operates with a red reference beam at 650 nm and a UV light at 375 nm (Cerovic et al., 2012). This sensor produces relative measures of chlorophyll (a + b), flavonoid and anthocyanin contents, and calculates the nitrogen balance index (NBI), which is the ratio of chlorophyll/flavonoids related to the nitrogen and carbon allocation. It is a nitrogen plant status indicator that is directly correlated with nitrogen mass content and therefore to the availability of N, and it is less sensitive to the variations in leaf age and leaf thickness than the chlorophylls (Cerovic et al., 2012). For each plot, measurements were carried out on the adaxial side of three random upper leaves. Three biological replicates per genotype and treatment were performed.

#### 2.2.2 Relative water content

Twelve days after initiation of stress treatment, RWC was measured on fully expanded upper third or fourth leaves. Three biological replicates were collected from separate plants. The fresh weight of harvested leaflets was immediately measured prior to incubation in distilled water overnight at 4 °C. Excess water was removed by blotting with tissue paper and the turgid weight was recorded. Turgid leaf samples were dried in an oven at 70 °C for 24 h. Finally, dried leaf sample weights were recorded. RWC values of genotypes were calculated using the following equation (Barrs and Weatherleyt, 1962).


$$\begin{array}{c} RWC(\%):[(Fresh\;weight-Dry\;weight)/(Turgid\;weight\\ -Dry\;weight)]*100 \end{array}$$

#### 2.2.3 Stomatal conductance

Stomatal conductance (gs, mmol H_2_O m^−2^ s^−1^) was measured at 22, 26, 28, 34 and 36 DAP, using a porometer (SC-1, METER Group, Inc., Pullman, WA, USA) on the adaxial side of the third fully expanded leaf from the top of each plant. Three biological replicates were measured per genotype and treatment. Measurements were conducted between 09:00 and 11:00 AM to minimize circadian effects.

#### 2.2.4 Stable carbon and nitrogen signatures

Stable carbon (13C/12C) and nitrogen (15N/14N) isotope ratios, together with the total nitrogen content, were determined. Measurements of carbon and nitrogen isotopes were conducted performed on the mass spectrometry unit at UMR FARE INRAE Reims (Euro-EA elemental analyzer from Eurovector, Milano, Italia, and Delta Advantage isotope mass spectrometer from Thermo-Electron, Bremen, Deutchland). Isotopic results were expressed in standard δ –notation (Coplen, 2011).


$$X\;=[(\frac{Rsample}{Rstandard}-1)]x\;100$$

where X is the δ^13^C or δ^15^N value, and R is the 13C/12C or 15N/14N ratios, respectively. The δ^13^C values were reported relative to the Vienna PeeDee Belemnite standard, whereas the δ^15^N values were reported relative to the standard N2 in air (Farquhar et al., 1989).

#### 2.2.5 Remote sensing indices

One digital Red-Green-Blue (RGB) picture was taken per plot, holding the camera at 1.5 m above the plant canopy, in a zenithal plane and focusing near the center of each plot. Photographs were taken with a Nikon B500 camera. The camera had a set focal length of 35 mm, shutter speed of 1/1500 sec without flash, the aperture set to automatic, and the images were saved in JPEG format with a size of 1615 × 1520 pixels. Pictures were subsequently analyzed with the open source Breedpix 0.2 software designed for digital photograph processing of different color properties (Casadesús et al., 2007).This software enabled the determination of the RGB vegetation indices green area (GA) and greener area (GGA). Both are formulated based on the number of green pixels in the image, but differ due to GGA excluding yellowish-green tones and therefore more accurately describing the amount of photosynthetically active biomass and leaf senescence.

Eco physiological parameters are measured several times before and after irrigation is stopped, except RWC and carbon isotope composition. The average amplitude of each parameter was calculated.


$$\text{Δ}Parameter=\frac{\sum (X_{WR}-X_{C})}{n}$$

Where $X_{WR}$ was the mean of the parameter under stress conditions and $X_{C}$ was the parameter under control conditions and the n was the number of measurements.

### 2.3 Morphological and agronomical traits

After the drought period, the plant stem length and number of stems were recorded. The aerial parts of the plants were then dried in an oven at 80 °C for at least 48 hours, until a constant dry weight was achieved. The fresh tuber weight was measured, and the total number of tubers was counted. The roots were washed, their length measured, and then dried in the oven at 80 °C for at least 48 hours until a constant dry weight was reached.

Drought tolerance index (DTI) was estimated to assess the genotype response to drought as follows (Fernandez, 1992).


$$DTI=\;\frac{Y_{WR}\;\times Y_{C}}{{\left(Y_{CA}\right)}^{2}}$$

Where Y_WR_ was the average tuber weight under water restriction treatment, Y_C_ was tuber weight under control treatment, and Y_CA_ was the average of tuber weight of the eight genotypes under control treatment.

### 2.4 Statistical analysis

Statistical analysis was *performed* using the SPSS 21.0 statistical package (SPSS Inc., Chicago, IL, USA) and R (version 4.3.3). All reported experimental data were averages of three replicates of each variety and treatment for all traits and were subjected to analysis of variance (ANOVA). Tukey’s b test was used to determine the different significance levels among the factors. Pearson’s correlation was used to determine the relationship between the parameters. Significant differences with respect to each control were considered according to Tukey’s b test after ANOVA for each variety. *P < 0.05; **P < 0.01; ***P < 0.001.

Principal Component Analysis (PCA), heatmaps, and regression analyses were conducted in R using the FactoMineR, ggplot2, and pheatmap packages.

## 3 Results

### 3.1 Tuber weight and crop growth parameters

**Figure 1:**
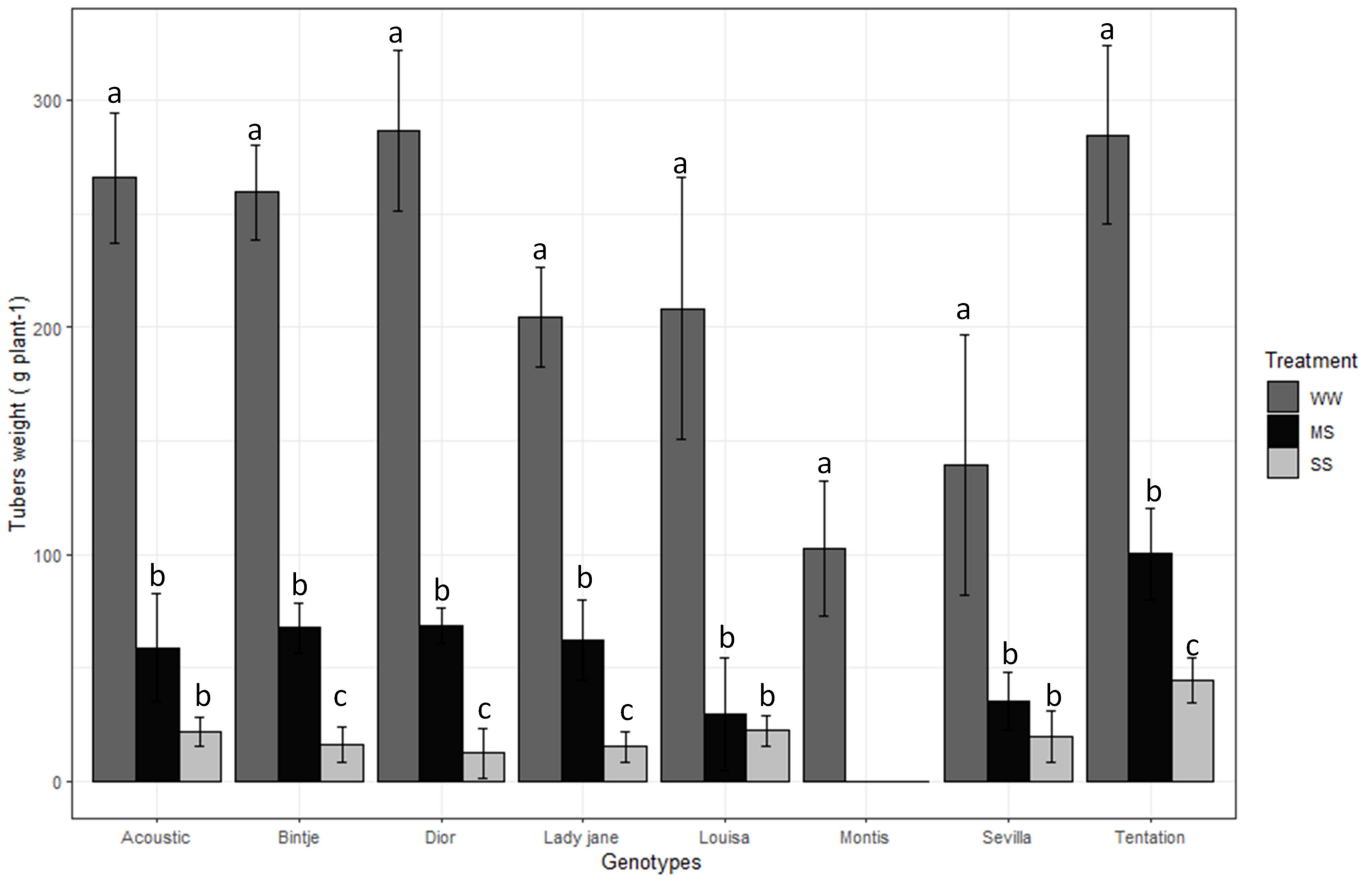
Average value standard error of tubers weight of eight genotypes under three irrigation treatments. Dark gray, Black and gray bars correspond to control well-watered (WW), moderate water stress (MS) and severe water stress (SS),respectively. Different letters indicate differences by using Tukey’b test at p<0.05.

Tuber weight was significantly affected by treatment, genotype, and their interaction (*P < 0.001*; **Table 2**). Across varieties and treatments, tuber weight ranged from 143.11 g plant^-1^ (Tentation) to 34.22 g plant^-1^ (Montis). On average, tuber yield across the eight varieties decreased by 75% under moderate waterstress (MS) and 91% under severe water stress (SS) compared to well-watered control (WW) conditions. Montis was the most affected variety (**Figure 1**), showing a complete reduction in tuber weight under both MS and SS, while Tentation exhibited the least reduction (64.76% under MS and 84.43% under SS).

**Table 2:** Mean values for Tuber weight, crop growth and root parameters of eight potatoes cultivar under different water regimes (WW, Control Well-Watered, MS, Moderate water stress, SS, severe water stress).

Cultivar	Tuber weight (g plant^-1^)	Ntubers		Biomass (g plant^-1^)	Plant height (cm)	Nstem	HI (%)	Root length (cm)	Root weight (g plant^-1^)
Acoustic	115.56^abc^	11.00^abc^		19.98^bc^	47.22^d^	3.56^c^	5.04^b^	62.33^a^	4.93^c^
Binjte	114.44^abc^	14.56^a^		21.77^b^	54.33^c^	6.00^b^	4.60^b^	42.78^de^	6.22^bc^
Dior	122.56^ab^	10.89^abc^		21.38^b^	53.22^c^	5.33^bc^	5.09^b^	39.56^e^	6.64^b^
Lady jane	94.11^bcd^	8.22^bc^		23.16^b^	61.44^b^	3.44^c^	3.62^b^	56.89^abc^	5.81^bc^
Louisa	86.78^cd^	9.56^bc^		21.15^b^	66.00^a^	4.11^bc^	3.75^b^	51.00^bcd^	4.97^c^
Montis	34.22^e^	3.56^d^		28.40^a^	52.33^c^	3.78^c^	0.83^c^	55.89^abc^	6.48^b^
Sevilla	64.67^d^	6.44^cd^		31.33^a^	51.56^c^	8.78^a^	1.84^c^	59.06^ab^	10.19^a^
Tentation	143.11^a^	11.33^ab^		17.46^c^	46.33^d^	4.56^bc^	7.72^a^	46.72^cde^	3.20^d^
WW	218.92^a^	15.08^a^		30.06^a^	56.46^a^	4.88	8.30^a^	63.54^a^	7.66^a^
MS	52.83^b^	7.75^b^		20.72^b^	48.83^b^	4.88	2.80^b^	48.54^b^	5.78^b^
SS	19.04^c^	5.50^c^		18.47^c^	49.21^b^	5.08	1.09^c^	43.25^b^	4.72^c^
G	0.000	0.000		0.000	0.000	0.000	0.000	0.000	0.000
T	0.000	0.000		0.000	0.000	ns	0.000	0.000	0.000
G * T	0.000	ns		0.000	ns	ns	0.001	ns	0.003

Means followed by different letters were significantly different by Tukey’s b test at *p* < 0.05. ANOVA factors: G, Genotype; T, Treatment; G × T, Genotype × Treatment interaction.

Growth parameters were significantly influenced by genotype (*P < 0.001*) for all traits and by treatment (*P < 0.001*) except for stem number (nStem). The genotype × treatment interaction was significant only for biomass and harvest index (HI; *P < 0.001*).

**Figure 2:**
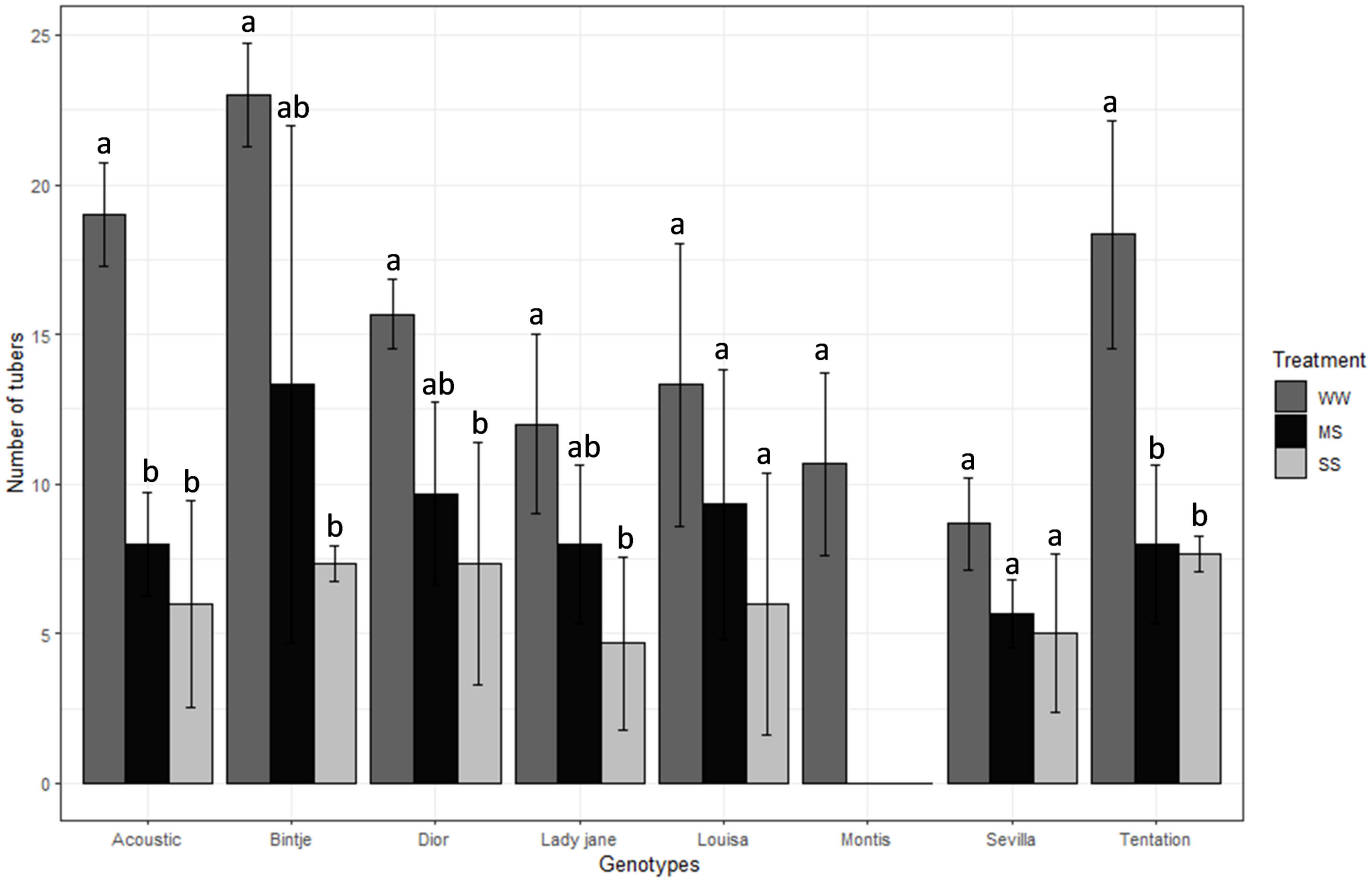
Average value standard error of tubers weight of eight genotypes under three irrigation treatments. Dark gray, Black and gray bars correspond to control well-watered (WW), moderate water stress (MS) and severe water stress (SS) respectively. Different letters indicate differences by using Tukey’b test at p<0.05.

nTubers ranged from 14.56 (Bintje) to 3.56 (Montis), and nStem ranged from 8.78 (Sevilla) to 3.44 (Lady Jane). Compared to WW, nTubers decreased by 48% under MS and 63% under SS (**Table 2**). Montis was the most affected variety (**Figure 2**) with a 100% reduction in nTubers under both MS and SS, while Binjte showed the least reduction in nTubers (42.04% under MS and 68.13% under SS).

**Figure 3:**
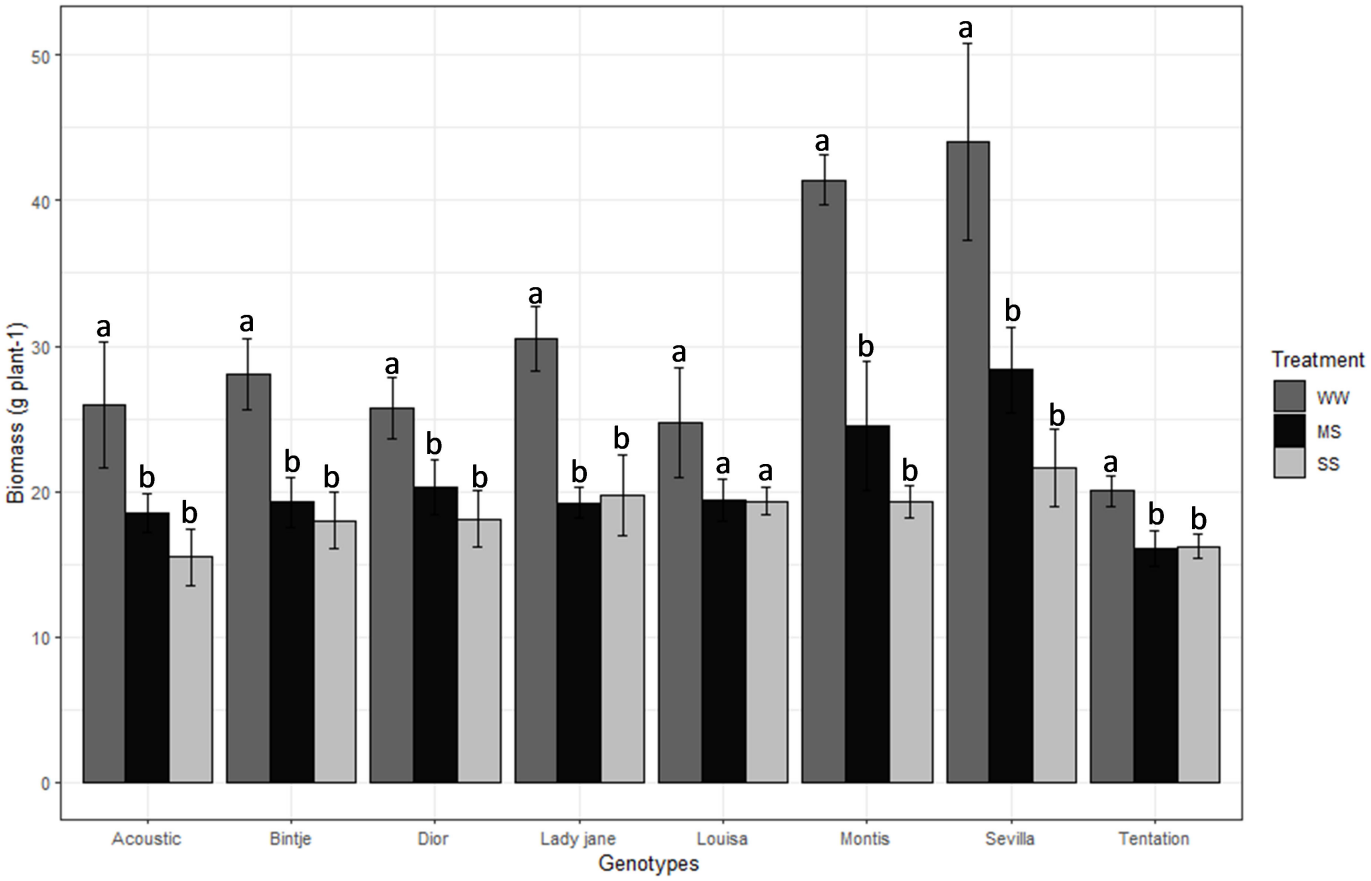
Average value standard error of Biomass of eight genotypes under three irrigation treatments. Dark gray, Black and gray bars correspond to control well-watered (WW), moderate water stress (MS) and severe water stress (SS) respectively. Different letters indicate differences by using Tukey’b test at p<0.05.

Biomass and Plant height ranged among varieties between 31.33 g (Sevilla) to 17.46 g (Tentation) and 66 cm (Louisa) to 46.33 cm (Tentation), respectively (**Table 2**). Biomass decreased by 31% and 58% for MS and SS, respectively, compared with WW (**Table 2**). Montis was the most affected variety in terms of biomass (**Figure 3**), showing a 40.84% reduction under MS and 53.51% under SS, while Tentation showed the least reduction in biomass (19.70% under MS and 19.00% under SS).

HI, root length and root weight ranged among varieties between 7.72% (Tentation) to 0.83% (Montis), 62.33 cm (Acoustic) to 39.56 cm (Dior) and 10.19 g plant^-1^ (Sevilla) to 3.20 g plant^-1^ (Tentation).

### 3.2 Water status and nitrogen parameters

δ^13^C (‰) was significantly affected by treatment (*P < 0.001*) and genotype (*P < 0.001*) but not by the genotype × treatment interaction (**Table 3**). Across treatments, δ^13^C values ranged from -28.1‰ (Acoustic) to -26.7‰ (Dior).

**Table 3:** Mean values for carbon isotope composition, water status and nitrogen isotope composition of eight potatoes cultivar under different water regimes (WW, Control Well-Watered, MS, Moderate water stress, SS, severe water stress).

Cultivar	δ^13^C (‰)	WU (g)	WUE (g g^-1^)	RWC	%n	δ^15^N (‰)
Acoustic	-28.1^c^	6853.2	0.015^b^	91.5	44.4^a^	1.54^b^
Binjte	-27.7^bc^	6684.0	0.017^ab^	78.2	41.0^ab^	0.72^bc^
Dior	-26.7^a^	6795.0	0.016^ab^	77.3	32.8^b^	0.98^bc^
Lady jane	-27.7^bc^	6412.3	0.015^b^	82.9	44.2 ^a^	1.86^b^
Louisa	-27.9^bc^	7326.3	0.012^b^	86.6	38.8^b^	1.10^bc^
Montis	-27.7^bc^	6985.4	0.007^c^	87.3	46.0 ^a^	3.03^a^
Sevilla	-27.2^ab^	6976.0	0.013^b^	85.0	35.7^b^	3.52^a^
Tentation	-27.4^abc^	6855.9	0.020^a^	84.9	42.4 ^a^	-0.05^c^
WW	-28.5^a^	12793.3^a^	0.021^a^	84.6^a^	40.6^b^	0.94
MS	-27.1^b^	4967.3^b^	0.013^b^	78.7^ab^	50.5^ab^	-0.14
SS	-26.9^b^	2822.5^c^	0.009^c^	89.3^b^	53.4^a^	3.97
G	0.000	ns	0.000	ns	0.032	0.000
T	0.000	0.000	0.000	0.012	0.004	0.000
G * T	0.138	ns	0.021	ns	ns	0.020

Means followed by different letters were significantly different by Tukey’s b test at *p* < 0.05. ANOVA factors: G, Genotype; T, Treatment; G × T, Genotype × Treatment interaction.

Water Use (WU, g) and Relative Water Content (RWC) were significantly affected by treatment (*P < 0.001*), with a decrease observed across treatments. WU ranged from 12793.3 g (WW) to 2822.5 g (SS). Under MS, RWC decreased slightly to 78.7%, and it increased slightly to 89.3% under SS (**Table 3**). And no significant differences were found between genotypes for either parameter.

Water Use Efficiency (WUE, g g^-1^) was also significantly influenced by treatment (*P < 0.001*). WUE values ranged from 0.021 g g^-1^ (WW) to 0.009 g g^-1^ (SS). Across treatments, excluding Montis (as it did not produce tubers under the water stress regime), Louisa exhibited the lowest WUE value (0.012 g g^-^¹), while Tentation (**Table 3**) showed the highest WUE value (0.020 g g^-^¹).

N (%) and δ^15^N (‰) varied among varieties, with N (%) ranging from 32.8 (Dior) to 46 (Montis) and δ^15^N(‰) ranging from 3.52‰ (Sevilla) to -0.05‰ (Tentation) (**Table 3**). For N (%), there was an increase of 24.4% in MS and 31.5% in SS compared to WW (**Table 3**). δ^15^N (‰) values were 0.94‰ in WW, -0.14‰ in MS, and 3.97‰ in SS. Significant effects were observed for both genotype and treatment for both parameters (**Table 3**).

### 3.3 Drought response of stomatal conductance, chlorophyll content, and flavonoid index for potatoes leaves with different days after planting

**Figure 4:**
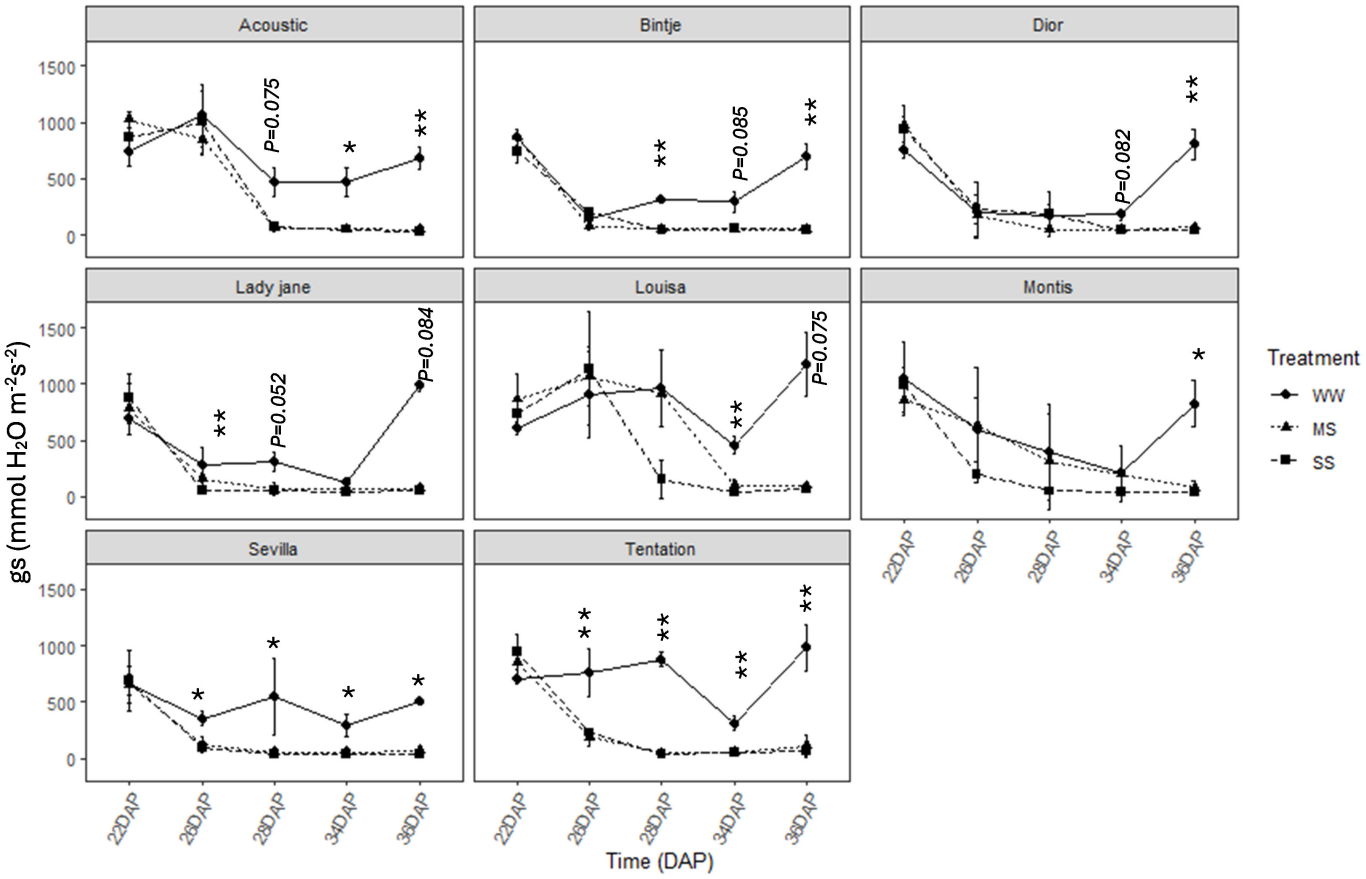
Average value +-standard error of stomatal conductance (gs) along the time (days after planting, DAP) in the eight genotypes under control and water restricted treatments. close circles, black triangles, black squares correspond to control well-watered (WW), moderate water stress (MS) and severe water stress (SS), respectively. Significant differences of ANOVA for each assessment are remarked with **p < 0.01, * p < 0.05.

The stomatal conductance (gs) was significantly decreased for all varieties (**Figure 4**) between 22 and 30 days after planting, with different varietal responses. Some varieties exhibited rapid stomatal closure under water stress while others maintained their stomata open for a long period. Seville and Tentation quickly closed their stomata under MS and SS with a marked decline observed in gs as early as 26 DAP, reaching low values 100 mmol H_2_O m^-2^ s^-1^. Louisa and Montis kept their stomata open for a longer period, showing a more gradual decrease in gs, with a minimum value recorded later (34 DAP).

**Figure 5:**
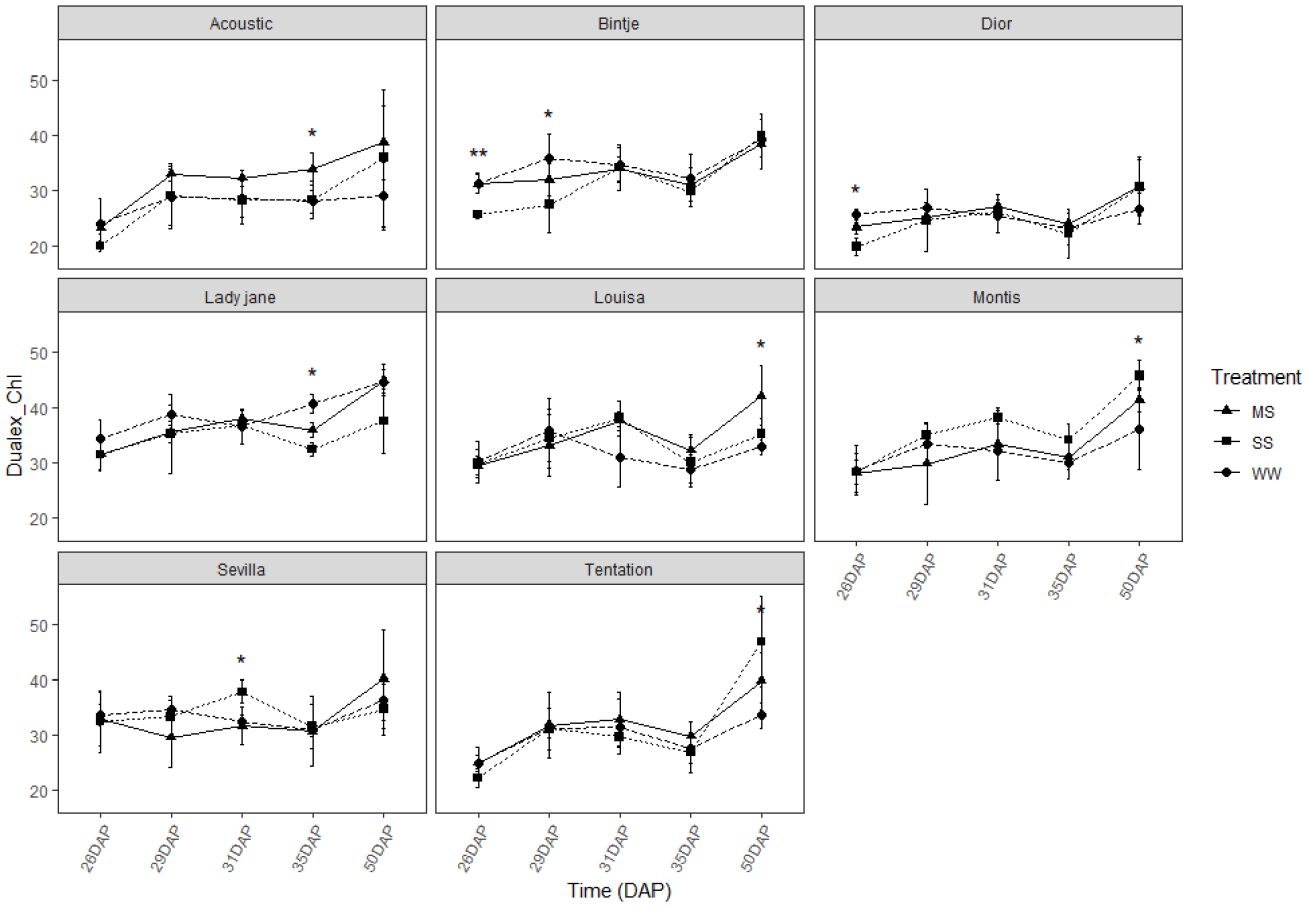
Average value ± standard error of Chlorophyll index (Dualex) along the time (days after planting, DAP) in the eight genotypes under control and water restricted treatments. Close circles, close triangle and close square correspond to control well-watered (WW), moderate water stress (MS) and severe water stress (SS), respectively. Significant differences of ANOVA for each assessment are remarked with ** p<0.01, * p<0.05.

For all varieties, the chlorophyll content measured using Dualex did not show significant differences between control and stressed conditions (**Figure 5**). However, there was a slight tendency for higher chlorophyll levels under stress conditions compared to the control.

**Figure 6:**
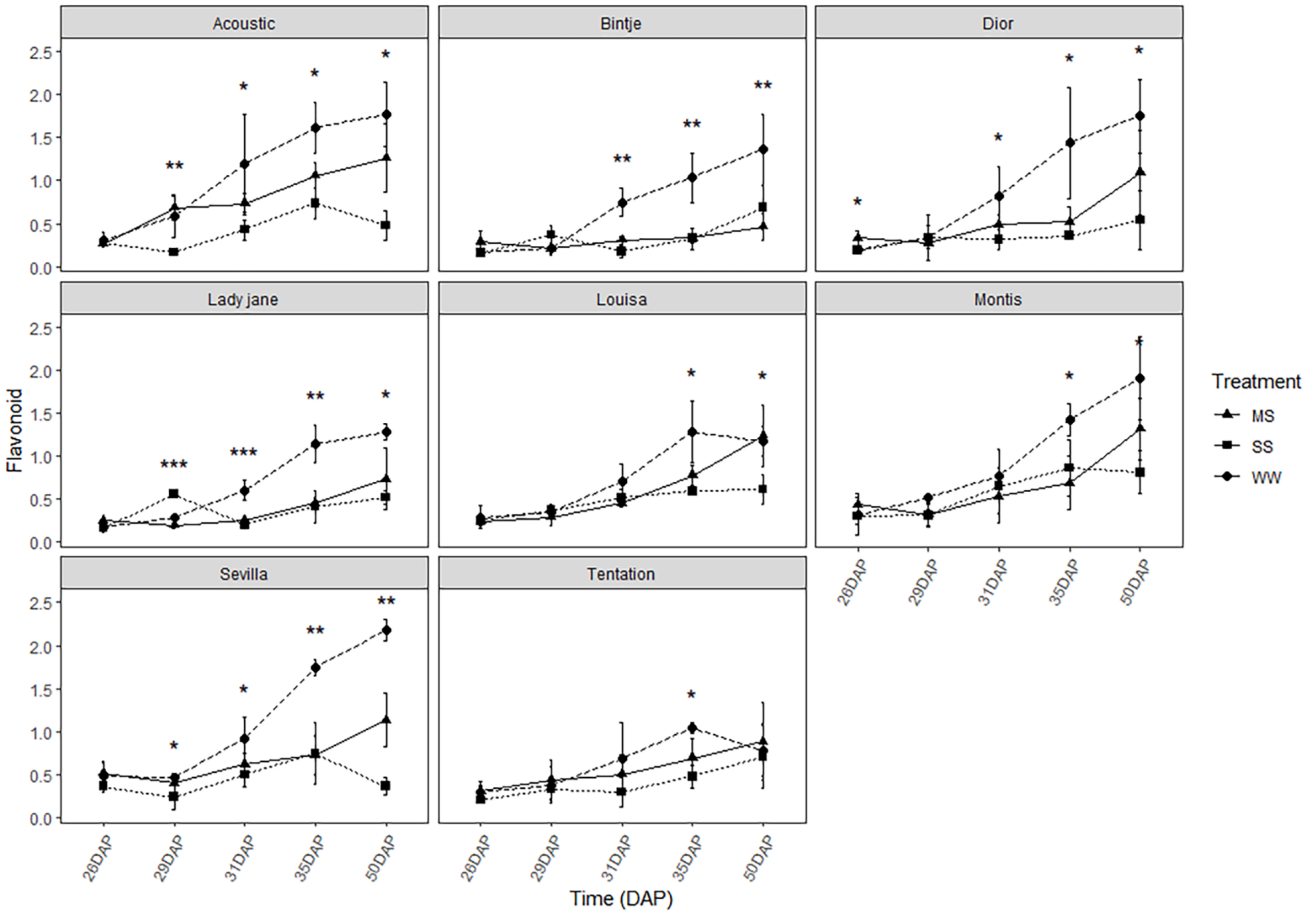
Average value ± standard error of Flavonoid index (Dualex) along the time (days after planting, DAP) in the eight genotypes under control and water restricted treatments. Close circles, close triangle and close square correspond to control well-watered (WW), moderatewater stress (MS) and severe water stress (SS), respectively. Significant differences of ANOVA for each assessment are remarked with ** p<0.01, * p<0.05.

The flavonoid index was significantly decreased for all varieties (**Figure 6**). All varieties showed a rapid decrease in the flavonoid index between 29 and 31 DAP, except for Tentation, Montis, and Louisa, which maintained the same level of flavonoids for a longer period. For Tentation, the decrease is less pronounced.

### 3.4 Correlation analysis of physiological parameters under moderate water stress and severe water stress

**Figure 7:**
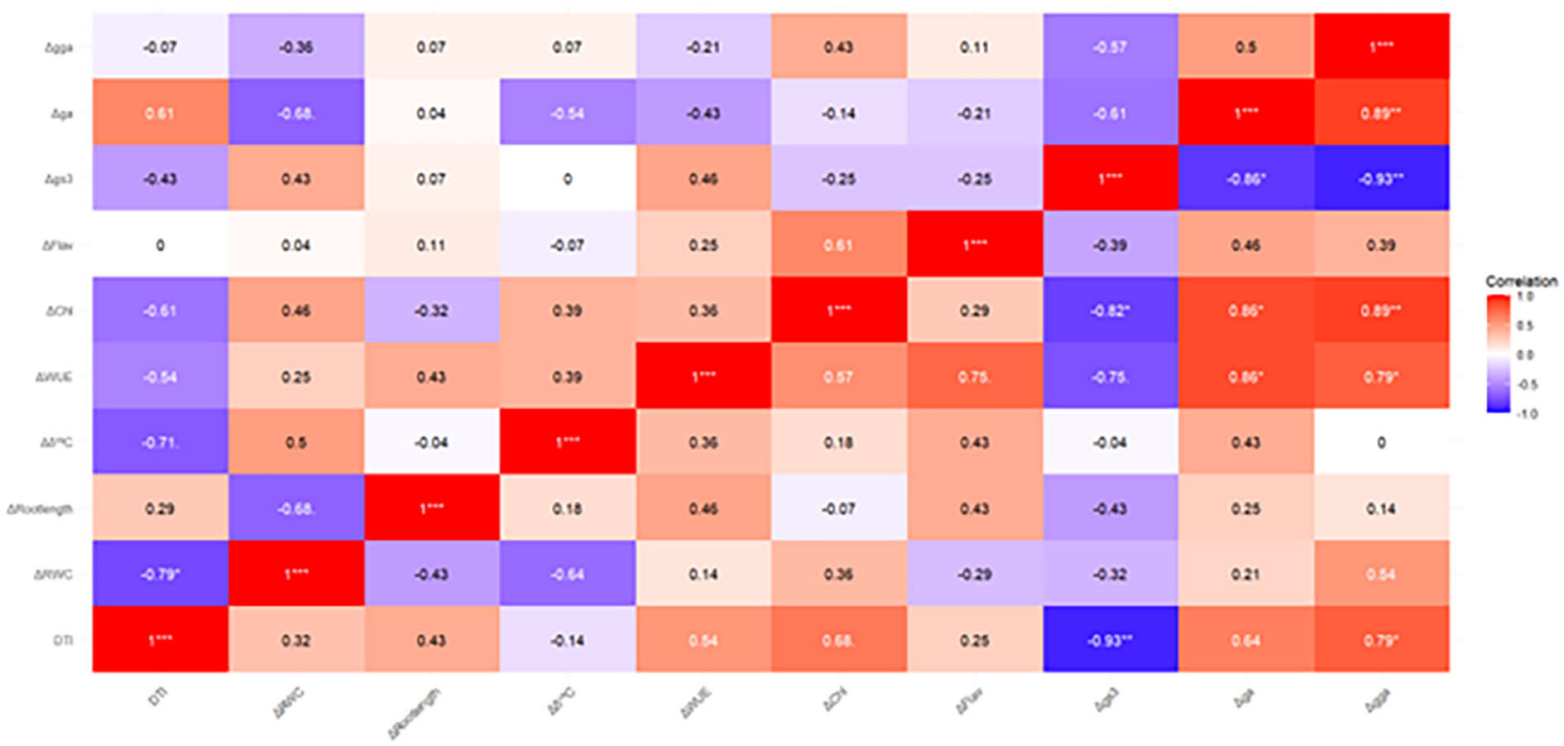
Pearson’s correlation coefficients between Drought Tolerance Index (DTI) and the average amplitude of physiological parameters [Relative Water Content (RWC), carbon isotope composition, Water Use Efficiency (WUE), Chlorophyll content (Chl), Flavonoid Index (Flav), stomatal conductance (gs)], as well as Root length, RGB vegetation indices green area (GA) and greener area (GGA), estimated under MS (above the diagonal) and SS (below the diagonal).

The heatmap analysis (**Figure 7**) reveals significant correlations between the amplitudes of physiological parameters and the Drought Tolerance Index (DTI) under moderate water stress and severe water stress.

Under moderate water stress, Δ RWC (r = -0.79*) and Δδ^13^C (r = -0.71.) show a negative correlation with DTI, meaning that increased drought stress is associated with reduced water retention and carbon fixation. This highlights the importance of maintaining good water retention and efficient carbon fixation to improve drought tolerance. In contrast, stomatal conductance amplitude (Δ gs) shows a moderate negative tendency (r = -0.48), suggesting that partial stomatal closure helps conserve water while still allowing adequate photosynthesis for plant growth.

Under severe drought stress, these correlations become more pronounced. For example, the relationship between Δ GGA (r = 0.79*) and DTI become strong, emphasizing the importance of canopy greenness in drought resilience. This suggests that plants with greener canopies tend to be more drought-tolerant, likely due to better water management and biomass maintenance. Δ gs shows a stronger negative correlation (r = -0.93**), indicating that under severe stress, tight stomatal regulation is crucial to prevent excessive water loss. Additionally, chlorophyll content amplitude (Δ chl) shows a positive correlation (r = 0.68.), indicating that maintaining chlorophyll content in drought conditions helps sustain biomass, further enhancing drought tolerance.

### 3.5 Trait associated under drought

**Figure 8:**
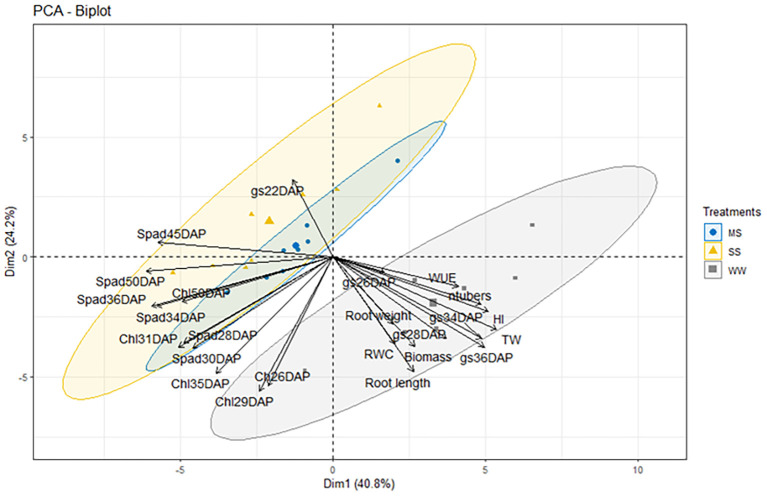
Principal component bi-plot scores PC1 vs PC2 showing the clustering of control well-watered (WW), moderate water stress (MS) and severe water stress (SS) plants, and the contribution of various traits to the variation in the dataset at different DAP. The traits are TW (Tubers Weight), ntubers (Numbers of Tubers), RL (Root Length), RW (Root Weight), HI(Harvest Index), Biomass, RWC (Relative Water Content), gs (Stomatal conductance) in different days after planting (22-26-28-34–36 DAP), SPAD (Chlorophyll content using SPAD) on different days after planting(28-30-35-45-50), Chl (Chl content using Dualex) on different days after planting(26-29-31-35–50 DAP).

The Principal Component Analysis (PCA) provided a comprehensive overview of the relationships among the measured physiological and agronomic parameters, enabling the identification of key traits driving variability across treatments and genotypes, particularly under drought conditions (**Figure 8**). The first principal component (PC1) explained 40.8% of the total variance, with strong positive loadings from traits such as total weight (TW), harvest index (HI), and late-stage stomatal conductance (gs34DAP, gs36DAP), which are closely associated with productivity and water use efficiency. These variables highlight the predominance of traits related to yield potential and stress tolerance in differentiating genotypes. In contrast, PC1 exhibited negative associations with chlorophyll indices (e.g., Spad28DAP, Spad36DAP, Chl31DAP), suggesting an inverse relationship between photosynthetic pigment retention and yield-related traits, potentially reflecting adaptive responses to drought stress.

The second principal component (PC2) accounted for 24.2% of the variance, primarily capturing root system traits and early physiological responses under water deficit conditions. High negative loadings from root length (RL), chlorophyll concentration (Chl29DAP, Ch26DAP), and related parameters emphasize the role of root activity and photosynthetic efficiency under drought stress. Positive contributions from early stomatal conductance (gs22DAP) indicate a secondary axis of variability related to early water management strategies, such as the ability to maintain water uptake during the initial stages of drought.

Overall, the PCA revealed a clear separation of genotypes and treatments based on productivity, water use efficiency, drought tolerance, and root-system traits, providing insights into the complex trade-offs between yield, photosynthetic efficiency, and resource acquisition strategies under drought conditions. These findings underscore the value of PCA in understanding the multifactorial interactions influencing crop performance, particularly under stress environments like drought.

## 4 Discussion

Drought is one of the major abiotic stresses that limits potato production. Drought significantly impacts potato cultivation, reducing growth, yield, and yield components of potato. The current study evaluated the drought tolerance of eight potato genotypes at the early growth stage, based on physiological and morphological traits assessed under well-watered control and deficit irrigation conditions (moderate water stress and severe water stress). Deficit irrigation significantly reduced tuber weight, tuber number and Biomass (Gervais et al., 2021; Lahlou et al., 2003; Muthoni and Kabira, 2016; Sprenger et al., 2016), which highlight the crop’s sensitivity to water availability, due to its shallow root system and high transpiration rates (Obidiegwu et al., 2015; Zarzyńska et al., 2017). Under stressed environment, varieties significantly respond differently, demonstrating notable genetic diversity in drought resistance. Tentation showed the greatest resilience, with only a 64.76% reduction in yield under moderate water stress, while Montis was the most vulnerable and did not produce any tubers under both stress levels. These variations indicate the presence of effective physiological mechanisms in certain genotypes, such as water conservation strategies, maintenance of photosynthetic structures, and resource allocation under restrictive conditions. These results highlight the significance of selecting or developing genotypes with enhanced drought resistance mechanisms to maintain yield under limited water availability, a situation anticipated to become more common due to climate change (Rauf et al., 2016).

Significant reductions in biomass, tuber number, and plant height under stress conditions reflected the general inhibition of vegetative development, likely due to decreased photosynthetic activity, nutrient limitation, and restricted cell division and elongation (Yang et al., 2021). Notably, the significant genotype × treatment interaction for biomass and harvest index (HI) indicates that some genotypes adjust their growth and carbon partitioning more effectively under drought. For instance, Tentation maintained relatively stable biomass under both stress levels, pointing to superior resource use efficiency and physiological plasticity. These findings align with (Mthembu et al., 2022)who reported that drought-tolerant potato genotypes exhibit higher water use efficiency under stress, making it a valuable selection trait. Isotopic analysis provided further insights into drought responses (Araus et al., 2012; Obidiegwu et al., 2015). The increase in δ¹³C values under stress conditions indicates reduced carbon isotope discrimination, likely due to stomatal closure aimed at minimizing water loss (Mateus and Lavres, 2025). This physiological adaptation, well-documented in drought studies (Farquhar et al., 1989), was particularly pronounced in genotypes such as Dior, reflecting higher intrinsic water use efficiency. Reductions in water use (WU) and relative water content (RWC) across treatments confirmed overall water stress effects, although some genotypes maintained higher RWC levels, possibly due to better stomatal regulation or deeper root systems.

The observed increase in nitrogen content (N%) under water stress and the wide range of δ^15^N values suggest a shift in nitrogen metabolism, potentially linked to reduced soil microbial activity or internal nitrogen remobilization. These changes reflect metabolic adjustments aimed at optimizing nitrogen use under stress (Gessler et al., 2004) which may vary across genotypes based on their drought adaptation strategies (Yan et al., 2023).

Stomatal conductance (gs) exhibited highly genotype-specific patterns, with cultivars such as Tentation and Sevilla closing their stomata quickly under stress, a typical response to conserve water under drought conditions (LE et al., 2011; Mateus and Lavres, 2025). The rapid stomatal closure may help to reduce water loss by increasing the water use efficiency and reducing the amount of water lost per Co_2_ molecule assimilate (LE et al., 2011). In contrast, Louisa and Montis maintained higher gs values for longer periods, which may lead to faster dehydration under prolonged stress. These variations suggest that the rate at which a genotype can close its stomata is a critical factor in determining its ability to conserve water under drought. Moreover, the ability to regulate conductance tightly is a key feature of drought-tolerant cultivars (Sprenger et al., 2016). Recent proteomic analyses also confirm that drought-tolerant potato genotypes activate specific molecular responses to maintain cellular function under water and nitrogen stress, further emphasizing the physiological importance of stomatal regulation (Meise et al., 2023).

Chlorophyll content remained relatively stable across conditions in all genotypes (**Figure 5**), indicating some resilience of the photosynthetic apparatus, although this does not necessarily equate to sustained photosynthetic performance, but this stability is a positive sign, indicating that the photosynthetic apparatus of the plants did not suffer significant damage under drought stress. However, a slight decrease in the flavonoid index was observed under stress for most varieties (**Figure 6**), suggesting that oxidative stress was occurring. Flavonoids are known to play a role in protecting plant cells from oxidative damage, and the reduction in flavonoid levels indicates that, in some varieties, the antioxidant defense mechanisms were overwhelmed under stress conditions. Interestingly, Tentation and Louisa maintained stable levels of flavonoids under stress (**Figure 6**), which may indicate a more robust antioxidant system capable of mitigating oxidative damage in response to drought stress.

The relationship between physiological parameters and Drought Tolerance Index (DTI) highlighted key traits associated with stress resilience. Under moderate water stress, strong negative correlations between DTI and both ΔRWC and Δδ¹³C emphasize the importance of maintaining tissue hydration and efficient carbon fixation for drought performance. Under severe stress, Δgs exhibited a very strong negative correlation with DTI (r = -0.93), suggesting that tight stomatal control is critical to prevent excessive water loss while supporting minimal gas exchange for growth. Additionally, a positive correlation between chlorophyll content amplitude (ΔChl) and DTI supports the notion that preserving photosynthetic pigments contributes to biomass retention and stress tolerance.

Principal Component Analysis (PCA) provided a comprehensive overview of how different physiological and agronomic traits are to drought tolerance. The first principal component (PC1), accounting for 40.8% of total variance, was dominated by yield-related traits such as tuber weight, HI, and late-stage stomatal conductance, identifying key features of genotypes that sustain productivity under stress. In contrast, PC1 showed negative associations with chlorophyll indices, implying a trade-off between pigment retention and yield potential. The second principal component (PC2) captured root traits and early physiological responses, including root length and early gs, underlining the role of early water uptake and adaptive root strategies. Genotypes that scored high on both PC1 and PC2 were those that combined good early water uptake with sustained productivity under stress, suggesting a comprehensive drought-resilience strategy that involves both early and late-stage physiological adjustments.

In our study, several traits emerged as particularly important for evaluating drought tolerance in potato. Yield-related parameters such as tuber weight, number of tubers, and harvest index (HI) were the most sensitive indicators of drought stress and therefore remain essential targets for breeding (Farooq et al., 2009; Gervais et al., 2021; Muthoni and Kabira, 2016). Among physiological traits, maintaining higher relative water content (RWC) and water use efficiency (WUE), together with tight stomatal regulation (Δgs), proved to be critical for sustaining growth under water-limited conditions (Alvarez-Morezuelas et al., 2022; Gervais et al., 2021). In addition, the preservation of chlorophyll content and canopy greenness (ΔChl, ΔGGA) contributed to maintaining photosynthetic activity and biomass accumulation under stress (Nasir and Toth, 2022; Rolando et al., 2015). Overall, our results indicate that genotypes combining efficient water conservation strategies, stable photosynthetic pigment levels, and sustained tuber production are more productive under drought, making this combination of traits a promising target for improving potato resilience to climate change.

Moreover, nutrient management may further enhance drought tolerance. Recent studies indicate that combined application of NPK, farmyard manure, and biofertilizers can improve potato growth, yield, and soil nutrient dynamics, even under water-limited conditions (Tiwari et al., 2025). This suggests that integrating optimized fertilization with drought-tolerant genotypes could provide a practical strategy to sustain productivity under water stress.

In conclusion, this study reinforces the complexity of drought tolerance in potatoes and emphasizes the importance of selecting cultivars with a combination of morphological, physiological, and biochemical traits that can withstand water stress. The differences observed between genotypes in terms of stomatal regulation, water use efficiency, and antioxidant response suggest that a multi-trait approach should be adopted for breeding programs aimed at improving drought tolerance. As climate change continues to exacerbate water scarcity, developing drought-resistant potato varieties will be crucial for ensuring food security and sustaining potato production in vulnerable regions.

## Data Availability

The raw data supporting the conclusions of this article will be made available by the authors, without undue reservation.

## References

[B1] Ahmadi S. H. Andersen M. N. Plauborg F. Poulsen R. T. Jensen C. R. Sepaskhah A. R.. (2010). Effects of irrigation strategies and soils on field-grown potatoes: Gas exchange and xylem [ABA. Agric. Water Manage. 97, 1486–1494. doi: 10.1016/j.agwat.2010.05.002

[B2] Aliche E. B. Oortwijn M. Theeuwen T. P. J. M. Bachem C. W. B. Visser R. G. F. van der Linden C. G. (2018). Drought response in field grown potatoes and the interactions between canopy growth and yield. Agric. Water Manage. 206, 20–30. doi: 10.1016/j.agwat.2018.04.013

[B3] Alvarez-Morezuelas A. Barandalla L. Ritter E. Lacuesta M. Ruiz de Galarreta J. I. (2022). Physiological response and yield components under greenhouse drought stress conditions in potato. J. Plant Physiol. 278, 153790. doi: 10.1016/J.JPLPH.2022.153790, PMID: 36130414

[B4] Anithakumari A. M. Nataraja K. N. Visser R. G. F. van der Linden C. G. (2012). Genetic dissection of drought tolerance and recovery potential by quantitative trait locus mapping of a diploid potato population. Mol Breeding. 30, 1413–1429. doi: 10.1007/s11032-012-9728-5, PMID: 23024597 PMC3460171

[B5] Araus J. L. Serret M. D. Edmeades G. O. (2012). Phenotyping maize for adaptation to drought. Front. Physiol. 3. doi: 10.3389/FPHYS.2012.00305, PMID: 22934056 PMC3429076

[B6] Arve L. E. Torre S. Olsen J. E. Tanino K. K. (2011). “Stomatal responses to drought stress and air humidit,” in Abiotic stress in plants - mechanisms and adaptations. doi: 10.5772/24661

[B7] Barrs H. D. Weatherleyt P. E. (1962). A re-examination of the relative turgidity technique for estimating water deficits in leaves. Aust. J. Biol. Sci. 15, 413–428. doi: 10.1071/BI9620413

[B8] Casadesús J. Kaya Y. Bort J. Nachit M. M. Araus J. L. Amor S.. (2007). Using vegetation indices derived from conventional digital cameras as selection criteria for wheat breeding in water-limited environments. Ann. Appl. Biol. 150, 227–236. doi: 10.1111/J.1744-7348.2007.00116.X;WGROUP:STRING:PUBLICATION

[B9] Cerovic Z. G. Masdoumier G. Ghozlen N.B. Latouche G. (2012). A new optical leaf-clip meter for simultaneous non-destructive assessment of leaf chlorophyll and epidermal flavonoids. Physiol. Plant. 146, 251–260. doi: 10.1111/j.1399-3054.2012.01639.x, PMID: 22568678 PMC3666089

[B10] Coplen T. B. (2011). Guidelines and recommended terms for expression of stable-isotope-ratio and gas-ratio measurement results. Rapid Commun. Mass. Spectromet. 25, 2538–2560. doi: 10.1002/RCM.5129, PMID: 21910288

[B11] Farooq M. Wahid A. Kobayashi N. Fujita D. Basra S. M. A. (2009). Plant drought stress: Effects, mechanisms and management. Agron. Sustain. Dev. 29, 185–212. doi: 10.1051/AGRO:2008021

[B12] Farquhar G. D. Ehleringer J. R. Hubick K. T. (1989). Carbon isotope discrimination and photosynthesis. Annu. Rev. Plant Biol. 40, 503–537. doi: 10.1146/ANNUREV.PP.40.060189.002443

[B13] Fernandez G. C. J. (1992). “Effective selection criteria for assessing plant stress tolerance,” in Adaptation of food crops to temperature and water stress. Shanhua, Taiwan AVRDC. doi: 10.22001/WVC.72511

[B14] Gervais T. Creelman A. Li X. Q. Bizimungu B. De Koeyer D. Dahal K. (2021). Potato response to drought stress: physiological and growth basis. Front. Plant Sci 12. doi: 10.3389/fpls.2021.698060, PMID: 34456939 PMC8387673

[B15] Gessler A. Rennenberg H. Keitel C. (2004). Stable isotope composition of organic compounds transported in the phloem of European beech - Evaluation of different methods of phloem sap collection and assessment of gradients in carbon isotope composition during leaf-to-stem transport. Plant Biol. 6, 721–729. doi: 10.1055/S-2004-830350, PMID: 15570478

[B16] Haverkort A. J. Struik P. C. (2015). Yield levels of potato crops: Recent achievements and future prospects. Field Crops Res. 182, 76–85. doi: 10.1016/J.FCR.2015.06.002

[B17] Haverkort A. J. Verhagen A. (2008). Climate change and its repercussions for the potato supply chain. Potato Res. 51, 223–237. doi: 10.1007/s11540-008-9107-0

[B18] Intergovernmental Panel on Climate Change (IPCC, 2021). Climate Change 2021: The Physical Science Basis. Contribution of Working Group I to the Sixth Assessment Report of the Intergovernmental Panel on Climate Change. Cambridge University Press, Cambridge, United Kingdom and New York, NY, USA. doi: 10.1017/9781009157896"10.1017/9781009157896

[B19] Lahlou O. Ouattar S. Agronomie J.--. (2003). The effect of drought and cultivar on growth parameters, yield and yield components of potato. Science 23, 257–268. doi: 10.1051/agro:2002089i. Undefined.

[B20] Mateus N. S. Lavres J. (2025). Leaf carbon isotope composition: a key proxy for scaling up the best candidates. J. Exp. Bot. 76, 1491. doi: 10.1093/JXB/ERAF007, PMID: 40205631 PMC11981894

[B21] Monneveux P. Ramírez D. A. Khan M. A. Raymundo R. M. Loayza H. Quiroz R. (2014). Drought and heat tolerance evaluation in potato (*Solanum tuberosum L.)* . Potato. Res. 57, 225–247. doi: 10.1007/S11540-014-9263-3

[B22] Mthembu S. G. Magwaza L. S. Mashilo J. Mditshwa A. Odindo A. (2022). Drought tolerance assessment of potato (*Solanum tuberosum L.*) genotypes at different growth stages, based on morphological and physiological traits. Agric. Water Manage. 261, 107361. doi: 10.1016/J.AGWAT.2021.107361

[B23] Muthoni J. Kabira J. N. (2016). Potato production under drought conditions: identification of adaptive traits. Int. J. Horticult. 6, 1–10. doi: 10.5376/ijh.2016.06.0012

[B24] Nasir M. W. Toth Z. (2022). Effect of drought stress on potato production: A review. Agronomy 12. doi: 10.3390/agronomy12030635

[B25] Obidiegwu J. E. Bryan G. J. Jones H. G. Prashar A. (2015). Coping with drought: Stress and adaptive responses in potato and perspectives for improvement. Front. Plant Sci 6. doi: 10.3389/FPLS.2015.00542/XML, PMID: 26257752 PMC4510777

[B26] Rauf S. Al-Khayri J. M. Zaharieva M. Monneveux P. Khalil F. (2016). Breeding strategies to enhance drought tolerance in crops Vol. 2 (Springer, Cham: Springer), 397–445. doi: 10.1007/978-3-319-22518-0_11

[B27] Rolando J. L. Ramírez D. A. Yactayo W. Monneveux P. Quiroz R. (2015). Leaf greenness as a drought tolerance related trait in potato (*Solanum tuberosum L.*). Environ. Exp. Bot. 110, 27–35. doi: 10.1016/J.ENVEXPBOT.2014.09.006

[B28] Saravia D. Farfán-Vignolo E. R. Gutiérrez R. De Mendiburu F. Schafleitner R. Bonierbale M.. (2016). Yield and physiological response of potatoes indicate different strategies to cope with drought stress and nitrogen fertilization. Am. J. Potato. Res. 93, 288–295. doi: 10.1007/s12230-016-9505-9

[B29] Shoukhat S. Tutail A. (2025). APPLIED AGRICULTURE SCIENCES. Appl. Agric. Sci. 3, 1–8. doi: 10.25163/agriculture.3110053

[B30] Sprenger H. Kurowsky C. Horn R. Erban A. Seddig S. Rudack K.. (2016). The drought response of potato reference cultivars with contrasting tolerance. Plant Cell Environ. 39, 2370–2389. doi: 10.1111/pce.12780, PMID: 27341794

[B31] Tiwari D. Kumar S. Dubey D. Sachan S. Singh J. Kumar S.. (2025). Effect of NPK and fym with biofertilizer on quality of potato and soil nutrient dynamics. J. Global Innov. Agric. Sci. 13, 431–437. doi: 10.22194/JGIAS/25.1629

[B32] Vasquez-Robinet C. Mane S. P. Ulanov A. V. Watkinson J. I. Stromberg V. K. De Koeyer D.. (2008). Physiological and molecular adaptations to drought in Andean potato genotypes. J. Exp. Bot. 59, 2109–2123. doi: 10.1093/jxb/ern073, PMID: 18535297 PMC2413284

[B33] Yan W. Qin J. Jian Y. Liu J. Bian C. Jin L.. (2023). Analysis of potato physiological and molecular adaptation in response to different water and nitrogen combined regimes. Plants 12, 1671. doi: 10.3390/PLANTS12081671/S1, PMID: 37111894 PMC10145361

[B34] Yang X. Lu M. Wang Y. Wang Y. Liu Z. Chen S. (2021). Response mechanism of plants to drought stress. Horticulturae 7, 50. doi: 10.3390/HORTICULTURAE7030050

[B35] Zarzyńska K. Boguszewska-Mańkowska D. Nosalewicz A. (2017). Differences in size and architecture of the potato cultivars root system and their tolerance to drought stress. Plant. Soil Environ. 63, 159–164. doi: 10.17221/4/2017-PSE

